# Draft Genome Sequence of *Dichelobacter nodosus* ATCC 25549, Strain VPI 2340 [11342], a Bacterium Causing Footrot in Sheep

**DOI:** 10.1128/genomeA.01002-15

**Published:** 2015-09-24

**Authors:** Alexandra Jackson, María Victoria Humbert, Anish Pandey, Holly Bratcher, Myron Christodoulides

**Affiliations:** a Neisseria Research Laboratory, University of Southampton Faculty of Medicine, Southampton, United Kingdom; b Molecular Microbiology, Academic Unit of Clinical and Experimental Sciences, Sir Henry Wellcome Laboratories, University of Southampton Faculty of Medicine, Southampton, United Kingdom; c Department of Zoology, University of Oxford, Oxford, United Kingdom

## Abstract

We report a draft genome sequence for *Dichelobacter nodosus* ATCC 25549, strain VPI 2340 [11342], a causative agent of ovine footrot. The draft genome shares ~98% gene similarity with the available genome of *D. nodosus* strain VCS1703A but is differentiated by extensive gene duplication and the absence of 13 particular genes.

## GENOME ANNOUNCEMENT

*Dichelobacter nodosus*, a Gram-negative, non-spore-forming obligate anaerobe, is the primary causative organism of footrot in ungulates (mammals with hooves, including cattle, sheep, deer, and goats) (1). The most well-known disease caused by *D. nodosus* is ovine footrot, a highly contagious necrotic disease of sheep hooves (2). A secondary role is postulated for *Fusobacterium necrophorum* through synergistic interactions with *D. nodosus* in footrot lesions (3). Knowledge of the genetic diversity and epidemiology of *D. nodosus* in ungulates is needed, given the significant animal welfare implications and economic costs associated with the disease. However, there is a significant lack of publically available complete genome sequences for *D. nodosus*.

*D. nodosus* (Beveridge, 1941) ATCC 25549, strain VPI 2340 [11342] (4) was grown for 7 to 10 days on Eugon Agar with defibrinated sheep blood (5% vol/vol) at 37°C in an anaerobic gas jar with the Anaerogen gas-generating system (Oxoid, United Kingdom). Genomic DNA was extracted using a standard phenol-chloroform method, suspended in sterile ultrahigh-quality water (20 ng/µl), and purity confirmed by agarose (0.7% wt/vol) gel electrophoresis. DNA was sequenced using an Illumina HiSeq 2500 machine (Oxford Genomics Centre, Wellcome Trust Centre for Human Genetics, University of Oxford). The 150-bp short-read paired-end data were assembled with the *de novo* assembly algorithm Velvet (5) (version 1.2.08) combined with the VelvetOptimiser script (version 2.2.4). The minimum output contig size was set to 200 bp with the scaffolding option switched off; all other program settings were left at default. No read trimming was performed. The draft genome and annotations are available on the rMLST genome database (identification [ID] no. 121826) (http://pubmlst.org/rmlst/) (6), which runs on the Bacterial Isolate Genome Sequence Database (BIGSdb) platform (7). Comparative genome analysis was run with the Genome Comparator tool implemented within BIGSdb using the gene-by-gene analysis approach (8).

The draft *D. nodosus* (Beveridge, 1941) ATCC 25549 genome has 36 contigs in its assembly, and the total length is 1,452,418 bp, coding for 1,271 nonredundant genes (with an additional 7 incomplete). This draft genome sequence was compared to the genome sequence available for *D. nodosus* strain VCS1703A (9), of total length 1.39 Mb, encoding 1,276 genes (http://www.ncbi.nlm.nih.gov/nuccore/NC_009446.1). There are 1,263 identical genes (~98%) in both strains, but 13 genes in *D. nodosus* ATCC 25549—encoding virulence-associated protein (Vap)F, VapA′, VapI, RTX family protein, plasmid maintenance system killer family protein, an uncharacterized fimbrial protein, an uncharacterized membrane protein, and six hypothetical proteins—are absent. A comparative genome sequence study of 103 *D. nodosus* ovine isolates also showed high conservation (>95% sequence similarity) (10), but none of these sequences are available publicly. The *D. nodosus* ATCC 25549 genome contains 333 duplicated genes, and gene duplication is not observed in *D. nodosus* VCS1703A. Duplicated genes encode proteins associated with metabolism, transport, and efflux processes, outer membrane, and virulence, including porin(s), OmpA, proteases, VapB, VapG1, VapG2, VapG3, type IV fimbrial tip adhesin, and PilT. Increasing the number of available *D. nodosus* genome sequences, as well as the *F. necrophorum* coisolates, would provide insight into different virulence attributes and host-pathogen interactions and aid in the development of new vaccines.

### Nucleotide sequence accession numbers.

This whole-genome shotgun project has been deposited at DDBJ/EMBL/GenBank under the accession no. LGVW00000000. The version described in this paper is version LGVW01000000.
